# Assessing the Seasonal and Spatial Dynamics of Zooplankton through DNA Metabarcoding in a Temperate Estuary

**DOI:** 10.3390/ani13243876

**Published:** 2023-12-16

**Authors:** Jorge Moutinho, Diego Carreira-Flores, Pedro T. Gomes, Filipe O. Costa, Sofia Duarte

**Affiliations:** 1 Centre of Molecular and Environmental Biology (CBMA) and ARNET—Aquatic Research Network, Department of Biology, University of Minho, Campus Gualtar, 4710-057 Braga, Portugal; diego.carreira@bio.uminho.pt (D.C.-F.); pagomes@bio.uminho.pt (P.T.G.); fcosta@bio.uminho.pt (F.O.C.); 2 Institute of Science and Innovation for Bio-Sustainability (IB-S), University of Minho, 4710-057 Braga, Portugal

**Keywords:** DNA metabarcoding, cytochrome c oxidase subunit I, hypervariable region V4 18S, zooplankton, biomonitoring, coastal ecosystems, Lima River estuary, non-indigenous species

## Abstract

**Simple Summary:**

The routine monitoring of zooplankton is difficult due to their small size and morphological ambiguity. Also, the eggs and larva of meroplankton resemble one another, and therefore it is challenging to identify their species. Alternatively, DNA-based tools can provide precise species identifications, regardless of the size or the developmental stage of a specimen. We developed a protocol for testing the potential of DNA metabarcoding for assessing the seasonal and spatial dynamics of zooplankton in a temperate estuary. Both the seasonal and spatial gradients influenced recovered richness, composition, and taxonomic distinctness, confirming the great aptitude of DNA metabarcoding for providing higher density monitoring and shedding new light on the composition and dynamics of complex zooplankton.

**Abstract:**

Zooplankton are key components of estuarine trophic networks. However, routine monitoring is hindered by the difficulty of morphology-based identification. DNA-based methods allow us to circumvent some of these hurdles, providing precise species identifications regardless of the taxonomic expertise of the investigator or the developmental stage of the specimens. However, the process is dependent on the completeness of the reference libraries. In this study, we sought to evaluate the potential of DNA metabarcoding to assess the seasonal (summer, autumn, and early spring) and spatial dynamics of zooplankton (four locations spanning ca. 6 km) in the Lima estuary (NW Portugal). Two genetic markers were used: the cytochrome c oxidase subunit I and the V4 hypervariable region of the ribosomal 18S rRNA genes. Overall, 327 species were recovered, and both markers displayed minute overlap (7% were detected with both markers). Species richness, composition, and taxonomic distinctness were majorly influenced by the season, with a declining tendency from summer (highest number of exclusive species, n = 74) to spring. Second to season, the taxa composition was influenced by spatial variation where the most downstream site displayed the highest number of exclusive species, n = 53. A total of 16 non-indigenous species were detected using metabarcoding, but only one (*Austrominus modestus*) has been documented out in the estuary. In conclusion, both the seasonal and spatial gradients influenced the recovered richness, composition, and taxonomic distinctness, confirming the great aptitude of DNA metabarcoding for providing higher density monitoring and shedding new light on the composition and dynamics of complex zooplankton communities.

## 1. Introduction

Monitoring zooplankton diversity is crucial to assessing ecosystem health and the impacts of environmental changes. Most biomonitoring studies have documented the responses of macro-eukaryotes to environmental alterations [1,2,3], while mostly neglecting small zooplankton [4].

Zooplankton play a significant role in the dispersion and distribution of energy between lower trophic levels, such as bacteria and eukaryotic phytoplankton, and higher trophic levels (macrofauna); this is known as bottom-up control; thus, they play a key role in the carbon biogeochemical cycle of coastal ecosystems [5,6,7,8,9]. Because zooplankton are a key component within aquatic trophic networks, monitoring their community will provide important information including the composition of holoplankton and meroplankton (eggs and larvae of benthic and nektonic organisms). From that information, we can assess the trophic state of the zooplankton community as it undergoes seasonal changes. Ultimately, we will improve our understanding of how climate change impacts marine and coastal ecosystems [10,11,12,13,14]. For instance, these communities have been reported to be sensitive to changes in water levels of reservoirs, trophic changes, and water quality, where commonly used WFD metrics were found to be insufficient for comprehensive quality assessments in these locations [15]. Similarly, in several estuarine systems, zooplankton were found to quickly respond to environmental conditions and anthropogenic pressures [16,17,18,19]. In addition, meroplankton often represent a significant component of the zooplankton community; thus, they can influence the location and potential yield of pelagic fisheries, either as food or nurseries [20], which can be associated with estuaries and other transitional waters. Furthermore, an analysis of the meroplankton community may represent an indirect way to assess the benthic invertebrate and fish communities and their reproductive cycles [21], providing a unique dataset that is otherwise difficult to obtain.

Researchers have recognized that the zooplankton’s taxonomic composition is important to achieving an understanding of the links between the physical environment and higher trophic levels [14]. However, the morphological ambiguity of the early developmental stages (i.e., eggs, larvae, and juvenile stages) and the small size of zooplankton are serious challenges to the identification of their species based on morphology alone. Thus, the high phylogenetic diversity inherent in zooplankton samples, coupled with the effort needed to identify the specimens’ species, limits research. On the other hand, DNA metabarcoding and high-throughput sequencing [22,23] have revolutionized the way that researchers characterize biodiversity in different types of ecosystems [23]. Indeed, it can greatly improve zooplankton identification by discriminating between morphologically similar species, the morphological plasticity of certain taxa, and overcoming the ambiguity of the early development stages. Further, DNA metabarcoding is a powerful tool that can be used to respond quickly to the needs of environmental managers; indeed, it already has been realized as a tool for large-scale biodiversity analysis [24,25], including the analysis of zooplankton [26,27]. Yet certain pitfalls are still present in metabarcoding schema: e.g., the lack of identification of individual life stages and the relative abundances of species [25,28]. Nevertheless, metabarcoding can display a high discrimination of spatial and temporal patterns in metazoan planktonic assemblages [27,29,30], and it can resolve hidden diversity: e.g., rare, low-abundant, and newly introduced taxa, as well as hard-to-identify meroplankton [31,32,33,34,35]. More recently, the potential of using the metabarcoding approach to provide biomass estimates has been used with several taxonomic groups, albeit with different degrees of success [34,36,37,38]. Most metabarcoding studies on zooplankton biodiversity use a single molecular marker to recover diversity [31,32]; this limits taxonomic scope [39]. Still, several studies have employed multiple molecular markers and multiple primer sets, which have displayed their marker complementarity for screening zooplankton taxa [30,40,41].

Here we report a study that used multi-marker DNA metabarcoding to monitor zooplankton in the Lima River estuary, a coastal ecosystem located in northwest Portugal. Specifically, we assessed both the seasonal and spatial variations of zooplankton and identified non-indigenous species.

## 2. Materials and Methods

### 2.1. Description of the Study Area

**Figure 1:**
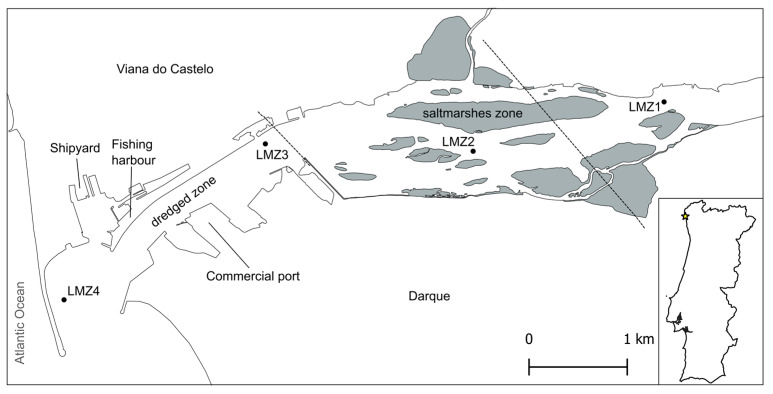
Sampling sites located in the Lima estuary, in the vicinity of Viana do Castelo, in the northwest of Portugal. On the map, saltmarshes are represented in grey and both bridges crossing the estuary are represented as dashed lines.

The Lima River estuary is temperate and drains into the Atlantic Ocean in the vicinity of the city of Viana do Castelo, in the northwest of Portugal (41°40′ N and 8°50′ W). It is characterized by a semidiurnal and mesotidal regime (3.7 m), with an average flushing rate of 0.40 m s^−1^, river flow of 70 m^3^ s^−1^, and hydraulic residence of nine days [16,42,43]. Due to the human presence on the shores, it has a quite consistent width of around 400 m; however, the upstream part is shallower and wider, reaching a maximum of 1 km in width [44]. The present study was conducted throughout the estuary and four sites were selected: LMZ1, LMZ2, LMZ3, and LMZ4 (Figure 1, Table S1). LMZ1 was the most upstream site. Located at the mouth of the river, LMZ4 was the most downstream. These two sites are ~6 km apart. In between the upper and lower reaches are two sites that have been highly modified by human intervention (LMZ3 and LMZ4). The lowermost part of the estuary encompasses several man-made structures and is subjected to the continuous dredging of the navigation channel [45]. Upstream from the Eiffel Bridge (close to LMZ3), the estuary has retained most of its natural banks, with shallow saltmarshes and tidal sandy islands (Figure 1). In general, the estuary receives industrial, agricultural, and urban discharges of nutrients and other materials [46].

### 2.2. Sampling Strategy

Sample collection took place during high tide in two different points of the estuary characterized by more predominant human activity (LMZ4: river mouth and LMZ3: recreational marina) and in two additional points located upstream of the saltmarshes (LMZ1 and LMZ2), where dredging activity is null, which limits boat traffic (Figure 1). Zooplankton was sampled using oblique tows and a standard plankton net with a 50 cm opening diameter, 150 cm length, and a mesh size of 153 µm. At each sampling location, three separate tows were performed, after which the end-cup content was poured into a storage bottle, previously washed with bleach (10%) and MilliQ water. Any considerable residual content inside the end-cup was washed into the bottle with water from the respective site. This process was conducted across three different seasons: summer (27 July 2021), autumn (17 November 2021), and spring (24 May 2022), resulting in a total of 36 zooplankton samples. The salinity, conductivity, and pH parameters were measured with a WTW Multiline F/set 3 no. 400327 (WTW, Weilheim, Germany) from superficial water samples (Table S1, Supplementary Material). The wave and wind conditions did not provide the right conditions to measure the water parameters from the river mouth during summer sampling.

All measured physical parameters displayed a decreasing tendency during the summer–spring period and throughout all sampled locations, although there were a few exceptions for LMZ3 and LMZ4. At the most downstream location (LMZ4), the salinity, conductivity, and pH increased considerably from autumn to spring. The highest values were attained at LMZ2 during the summer and autumn for all parameters, while in spring, the maximum values were found at LMZ4, the most downstream location (Table S1, Supplementary Material).

The zooplankton samples were stored in sterilized plastic flasks with water from their respective sampling site. The flasks were kept in a large container filled with ice until they reached the lab (2–3 h). When returned to the lab, we processed the samples immediately. Filtration was done through Merck-Millipore membranes (47 mm diameter, pore size of 45 µm) using an EZ-Fit™ Manifold filtration ramp equipped with three Microfil^®^ funnels attached to an EZ-Stream Vacuum Filtration Pump (Merck-Millipore, Darmstadt, Germany). All removable parts were submerged in bleach (10%) and rinsed with MilliQ water, and all surfaces of the working station were cleaned with bleach (10%) and ethanol (96%). To limit additional possibilities of cross-contamination between samples, we flamed the porous stone of the funnel base, which had previously been immersed in 96% ethanol. The filter membranes were then preserved at −20 °C until DNA extraction.

### 2.3. DNA Extraction, PCR Amplification, and High-Throughput Sequencing (HTS)

DNA was extracted from filtered samples using the DNeasy PowerSoil Kit, from Qiagen (Hilden, Germany), following the manufacturer’s protocol with minor changes. Two technical replicates were considered for each sample, consisting of ¼ of the scraped-off zooplankton from the filters. After extraction, and for each sample, the two technical replicates were pooled together (30 µL of each). Negative controls were introduced during this step using exactly the same procedure but with new filters for checking for any contamination of the solutions of the DNA extraction kits and labware materials used. These negative controls were further used during PCR reactions. DNA concentrations were quantified using a NanodropTM 1000 spectrophotometer (Thermo Fisher Scientific, Waltham, MA, USA) (Table S2, Supplementary Material) and stored at −20 °C until PCR amplification and high-throughput sequencing.

Samples were prepared for Illumina MiSeq sequencing by targeting the eukaryotic communities through the amplification of the 18S rRNA and COI genes at Genoinseq (Biocant, Cantanhede, Portugal). Two different sets of primers were used: the forward primer mICOIintF 5′-GGWACWGGWTGAACWGTWTAYCCYCC-3′ [47] with the reverse primer LoboR1 5′-TAAACYTCWGGRTGWCCRAARAAYCA-3′ [48], which targets the 3′ region of COI (~313 bp); and the forward primer TAReuk454FWD1 5′-CCAGCASCYGCGGTAATTCC-3′ with the reverse primer TAReukREV3 5′-CTTTCGTTCTTGATYRA-3′ [49], which targets the hypervariable region V4 (~380 bp) of the eukaryotic 18S rRNA gene. Both primers were selected based on previous marine invertebrate analysis [50,51], where the use of a 3′ COI fragment (hereafter mentioned as COI) provided more resolved and reliable species-level identifications, which were even greater and had higher success rates than the versatile primer sets traditionally used for DNA barcoding (i.e., LCO149/HCO2198) [47,48,50,51,52], whereas the hypervariable region V4 of the 18S rRNA gene (hereafter mentioned as 18S) provided a broader scope on the recovered biodiversity. Still, the 18S species-level identifications should be considered with caution, since species-level resolution can be poor in some groups [53,54]. Although the herein-employed primers have been rarely mentioned in metabarcoding-based assessments of zooplankton diversity, both markers have been widely employed in metabarcoding-based zooplankton assessments [47,48,51,55].

For both markers, PCR reactions were performed for each sample using a KAPA HiFi HotStart PCR Kit according to manufacturer suggestions, 0.3 μM of each PCR primer, and 5 μL (COI) and 2.5 μL of template DNA in a total volume of 25 μL. For the COI, PCR conditions involved a 3 min denaturation at 95 °C, followed by 35 cycles of 98 °C for 20 s, 60 °C for 30 s, and 72 °C for 30 s, as well as a final extension at 72 °C for 5 min. For the 18S, PCR conditions involved a 3 min denaturation at 95 °C, followed by 10 cycles of 98 °C for 20 s, 57 °C for 30 s, and 72 °C for 30 s, 25 cycles of 98 °C for 20 s, 47 °C for 30 s, and 72 °C for 30s, and a final extension at 72 °C for 5 min. Negative PCR controls were included for all amplification procedures. The negative control samples did not amplify for any primer pair.

The DNA was further reamplified in a limited-cycle PCR reaction to add sequencing adapters and dual indexes to both ends of the amplified target region according to the manufacturer’s recommendations (Illumina, San Diego, CA, USA). PCR products were then one-step purified and normalized using the SequalPrep 96-well plate kit (ThermoFisher Scientific, Waltham, MA, USA) [56], pooled and 250 bp paired-end sequenced in the Illumina MiSeq^®^ (50,000 sequencing depth) sequencer with the MiSeq reagent Kit v3 (600 cycles), according to manufacturer’s instructions (Illumina, San Diego, CA, USA) at Genoinseq (Cantanhede, Portugal). For further information regarding the amplification and sequencing steps, see Info S1 (Supplementary Material).

### 2.4. Bioinformatic Processing and Taxonomic Assignment

Quality filtration was performed on Illumina reads (fastq files) using PRINSEQ v0.20.4 [57] to remove sequencing adapters, trim bases with an average quality lower than Q25 in a window of 5 bases, and remove reads with less than 100 bases for 18S and 150 bases for COI. This initial processing was performed at Genoinseq.

Prior to taxonomic assignment, the filtered forward and reverse reads provided by the sequencing facility were merged by overlapping paired-end reads in mothur (*make.contigs* function, default) [58,59]. The resulting reads were then processed in two pipelines from public databases: the COI reads were submitted to the mBrave—Multiplex Barcode Research and Visualization Environment (www.mbrave.net; [60]; accessed on 19 December 2022) and the 18S reads were submitted to SILVAngs (ngs.arb-silva.de/silvangs/; [61]; accessed on 6 December 2022). In mBrave, the COI reads were trimmed by length (maximum 313 bp) and those with a minimum quality value (QV) higher than 10 were kept, which allowed for a maximum of 25% nucleotides with <20 QV and a maximum of 25% nucleotides with <10 QV. Reads were then taxonomically assigned using a similarity threshold of 97% against all the system’s reference libraries, the personally curated and Iberia Peninsula-specific reference libraries, as well as taxa-specific reference libraries (December 2022)—all from the BOLD database [62] (see Info S2 (Supplementary Material) for the list of the COI reference sequence libraries).

The 18S reads were processed by the amplicon analysis pipeline of the SILVA project (SILVAngs 1.4) [61]. Each read was aligned using the SILVA Incremental Aligner (SINA v1.2.10 for ARB SVN (revision 21008)) [63], against the SILVA SSU rRNA SEED, and quality controlled [61]. Reads shorter than 200 aligned nucleotides and reads with more than 1% ambiguities or 2% homopolymers, respectively, were excluded from further processing. Putative contaminations and artifacts, and reads with a low alignment quality (80 alignment identity, 40 alignment score reported by SINA) were identified and excluded from downstream analysis. After these initial steps of quality control, identical reads were identified (dereplication), the unique reads (100%) were clustered (OTUs) on a per-sample basis, and the reference read of each OTU was classified. Dereplication and clustering were done using VSEARCH (version 2.17.0; https://github.com/torognes/vsearch; accessed on 6 December 2022) [64], applying identity criteria of 1.00 and 0.99, respectively. The classification was performed by BLASTn (2.11.0+; http://blast.ncbi.nlm.nih.gov/Blast.cgi; accessed on 6 December 2022) [65], with standard settings using the non-redundant version of the SILVA SSU Ref dataset as classification reference (release 138.1; http://www.arb-silva.de; accessed on 6 December 2022). The classification of each OTU reference read was mapped onto all reads that were assigned to the respective OTU using a 99% similarity threshold (December 2022). For further detailed information regarding both bioinformatic pipelines (mBrave and SILVAngs), see Info S1 (Supplementary Material).

Any read assigned to a non-metazoan taxon was discarded. The nomenclature of detected taxa was confirmed using the World Register of Marine Species database (WoRMS; www.marinespecies.org, accessed on January of 2023). Additionally, all BINs (Barcode Index Numbers) to which COI reads were assigned were thoroughly curated (identification of misidentifications and synonymized species names associated with the same BIN, as well as ambiguous groupings) to attain the most reliable identifications, particularly at the species-level. Regarding the 18S dataset, sequences were blasted against the NCBI’s database to assess the reliability of the taxonomic assignment provided by the SILVAngs curated database [66]. Throughout further analysis and discussion, only species-level identifications were considered, due to the taxonomic uncertainty that can be associated with OTUs, while displaying more than 8 reads on each dataset [50,67].

### 2.5. Data Processing and Analysis

All statistics and graphics were performed with the Paleontological Statistics software (PAST, v4.09) and in the R environment (version 4.1.2.) using the Vegan package (version 2.6.4) [68], unless otherwise stated. Only presence/absence data was considered due to the putative amplification associated bias. Treemap was performed using the function *treemap* from the package of the same name [69] in order to present and determine the most relevant taxonomic groups recovered from the sampled zooplankton and further analyze the complementary effect of the multi-marker approach herein employed. 

Beyond this point, we only considered the merged COI and 18S datasets due to the complementarity of both markers in their species detection. Both taxonomic diversity and distinctness were determined using the PAST software, which determines both taxonomic metrics based on Clarke & Warwick [70]’s definition and the author’s own 1000 random replicates test, and can be represented by the following formula:

$$∆=\frac{\sum \sum_{i<j}w_{ij}x_{i}x_{j}}{\sum \sum_{i<j}x_{i}x_{j}},$$

where *w_ij_* are weights varying if concurrent species (0) or different species (1), and *x* are the abundances. Due to the nature of this study, both the taxonomic diversity and distinctness were represented with the same values (hereafter represented solely as taxonomic distinctness). Species richness and taxonomic distinctness fluctuations were assessed using a 2-way ANOVA, followed by post-hoc (Tukey’s HSD) analysis to assess the differences among the levels of each factor (season and location) using the *aov* and *TukeyHSD* functions, respectively. Non-metrical Multidimensional Scaling (nMDS) ordination was used to examine recovered community composition similarities among samples using Jaccard’s dissimilarity index. The influence of spatial/seasonal sampling was tested using the *adonis2* function (2-way PERMANOVA; 999 permutations). Prior to all multivariable analysis, the *decostand* function was used to transform read datasets into presence/absence, since the *metaMDS* (function used to perform nMDS) determines Jaccard’s dissimilarity based on Bray-Curtis [68]. The *cor* function was used to determine Pearson’s correlation of each species recovered with the dimensions used for the nMDS (n = 2), which was then used to determine the taxa that better fit the attained clustering, based on *p* value, using the *envfit* function. Furthermore, a presence–absence heatmap and clustering was performed—with the number of samples in which each taxon was recovered—using the *pheatmap* function of an R package of the same name [71], in order to assess the species-level clustering throughout the spatial and seasonal gradient. All previous analyses were repeated to analyze the most relevant taxonomic groups determined by the previous treemap analysis. 

All Venn diagrams, used for the visual representation of the partitioning of the recovered zooplankton diversity, were developed using the InteractiVenn platform [72].

## 3. Results

### 3.1. HTS Data Initial Processing and the General Taxonomic Composition Recovered by Each Marker

The high-throughput sequencing of the 36 samples resulted in a total of 2,096,992 and 1,548,889 recovered COI and 18S reads, respectively, from which, after filtration and quality checking, around 72% of COI reads were eligible for taxonomic assignment, but for 18S, less than half of the reads were eligible for taxonomic assignment (42%) (Table S3, Supplementary Material). Indeed, this was particularly observed in samples from the spring season collected at the most downstream location (LMZ4). 

**Figure 2:**
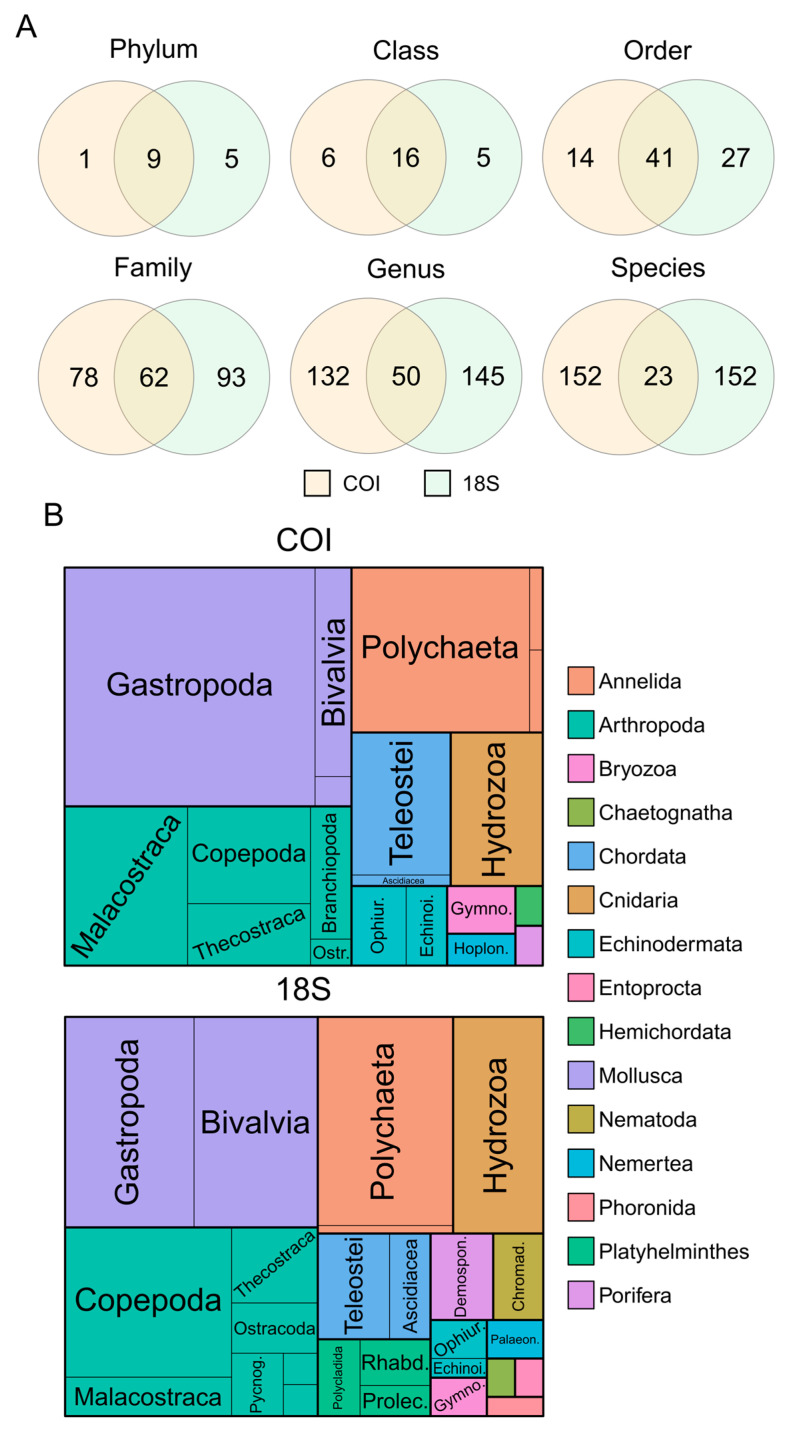
Partitioning of recovered taxa with COI and 18S (>97% and >99% similarity, respectively), depicted in all major taxonomic ranks (phylum, class, order, family, genus, and species) (**A**). Zooplankton composition, recovered through HTS using COI and 18S primers (**B**). The latter is color-coded based on recovered phyla. For further details, see the list of recovered taxa found in Table S4 (Supplementary Material).

Around 36.4 and 21.2% of COI and 18S reads, respectively, were taxonomically assigned to the aquatic metazoan. However, for a large portion of these, obtaining species-level assignments was not possible, resulting in their classification at higher taxonomic ranks. A total of 15 metazoan phyla were recovered (Figure 2A), from which Hemichordata was specifically recovered with COI, while Entoprocta, Chaetognatha, Nematoda, Platyhelminthes, and Phoronida were 18S-exclusive records (Figure 2B). Consequently, 18S dominated in the number of exclusive detections throughout the whole taxonomic spectrum (except for class and species ranks), particularly at the order level (almost two times greater than COI; Figure 2A). The 18S:COI ratio of exclusive taxonomic assignments was found to be lower the greater the taxonomic resolution obtained. On the other hand, markers’ complementarity was shown to be greater the higher the resolved taxonomic assignment (Figure 2A). Indeed, only 466,724 of the COI reads (ca. 22.3%) and 306,648 of the 18S reads (ca. 19.8%) were taxonomically assigned to species level, resulting in the detection of 175 species each, from which only 23 species (7%) were found to have been recovered with both markers (Figure 2A; for further details see Table S4, Supplementary Material), which represented less than half of the overlap observed at the genus level (Figure 2A).

From the total of 327 recovered species, 85% belonged to five different phyla: 49 and 55 species to Mollusca, 44 and 42 species to Arthropoda, 27 and 29 species to Annelida, 18 and 13 to Cnidaria, and 11 and 14 species to Chordata (for COI and 18S, respectively) (Figure 2B). The remaining recovered taxa represented 10 phyla, namely Bryozoa, Echinodermata, Nematoda, Nemertea, and Porifera, recovered with both markers, as well as Chaetognatha, Platyhelminthes, Phoronida, and Entoprocta 18S-exclusive, and Hemichordata COI-exclusive. The latter three phyla were solely represented by a singular species each. Arthropoda diversity was dominated by Copepoda (39%), followed by Decapoda (23%) and Thecostraca (15%). The 18S displayed greater taxonomic coverage of Copepoda than COI—18S recovered six different Copepoda orders (three exclusive: Monstrilloida, Polyartha, and Siphonostomatoida), as opposed to the three recovered with COI (Calanoida, Cyclopoida, and Harpacticoida). Both markers displayed greater representations of Calanoida during the study (61% and 69% of the Copepoda, for 18S and COI), followed by Harpacticoida (two 18S and three COI exclusive species) and Cyclopoida (two 18S and a COI exclusive species). Gastropoda (74% with COI and 34% with 18S) and Polychaeta (similar recovered richness with both markers) encompassed around 71.2% and 94.3% of the Mollusca and Annelida, respectively. Ascidiacea represented a fourth of the herein Chordata recovered, but most were detected by 18S. The remaining 10 phyla encompassed no more than a total of 10 species each (12.2%).

Because the two markers were complementary in species and taxonomic group detection, we opted to conduct the remaining analyses using the species-level dataset where data recovered with both markers were joined together, which allowed us to see more clearly the spatial and seasonal patterns of zooplankton in the Lima estuary.

Overall, around 32.4% of the recovered species consisted of single-sample recoveries, which included a greater representation of the overall Chordata and Porifera (72% and 60%), as well as half of the recovered Platyhelminthes, Nematoda, and Nemertea species. The only recovered Hemichordata species, *Balanoglossus clavigerus* (Delle Chiaje, 1829), was also detected in a single sample. On the other hand, only 28 species (ca. 8.6%) were recovered in more than 50% of the samples, with the majority being composed of Arthropoda (13 species) and Mollusca (9 species); however, only 13 species were recovered in the majority of samples (herein 75%), which accounted for nine Arthropoda species: the Calanoida *Temora longicornis* (Müller O.F., 1785) and *Pseudocalanus elongatus* (Brady, 1865), and the Thecostraca *Austrobalanus imperator* (Darwin, 1854), *Verruca stroemia* (O.F. Müller, 1776), *Austrominius modestus* (Darwin, 1854), *Chthamalus montagui* (Southward, 1976), *Amphibalanus improvisus* (Darwin, 1854), *Balanus trigonus* (Darwin, 1854), and *Sacculina carcini* (Thompson, 1836); three Mollusca species: the Gastropoda *Peringia ulvae* (Pennant, 1777), the Bivalvia *Hiatella arctica* (Linnaeus, 1767) and *Mytilus* sp., and the Hydrozoa *Obelia dichotoma* (Linnaeus, 1758).

### 3.2. Non-Indigenous Species Detection

DNA metabarcoding allowed the detection of 16 NIS, which accounted for around 4.9% of the recovered zooplankton biodiversity (Table S4, Supplementary Material). The bulk of recovered NIS were Arthropoda (7 species—*Amphibalanus eburneus* (Gould, 1841), *A. improvisus*, *A. modestus*, *B. trigonus*, *Eriocheir sinensis* (H. Milne Edwards, 1853), *Oithona davisae* (Ferrari F. D. & Orsi, 1984), and *Pseudodiaptomus marinus* (Sato, 1913)), followed by Ascidiacea (*Ciona intestinalis* (Linnaeus, 1767), *Microcosmus squamiger* (Michaelsen, 1927) and *Styela plicata* (Lesueur, 1823)), Mollusca (the gastropod *Crepidula fornicata* (Linnaeus, 1758), and the bivalves *Mercenaria mercenaria* (Linnaeus, 1758) and *Ruditapes philippinarum* (A. Adams & Reeve, 1850)). The remaining NIS included the polychaete *Pseudopolydora paucibranchiata* (Okuda, 1937), the bryozoan *Tricellaria inopinata* (d’Hondt & Occhipinti Ambrogi, 1985), and the hydrozoan *Cordylophora caspia* (Pallas, 1771). Overall, every recovered NIS consisted of exclusive detections by either the COI or the 18S (8 and 6 exclusive NIS recovered, respectively), except the copepod *P. marinus*, which was detected with both markers. For further details, see Table S4 (Supplementary Material).

### 3.3. Seasonal and Spatial Dynamic Effects on Species Richness, Taxonomic Composition, and Distinctness

**Figure 3:**
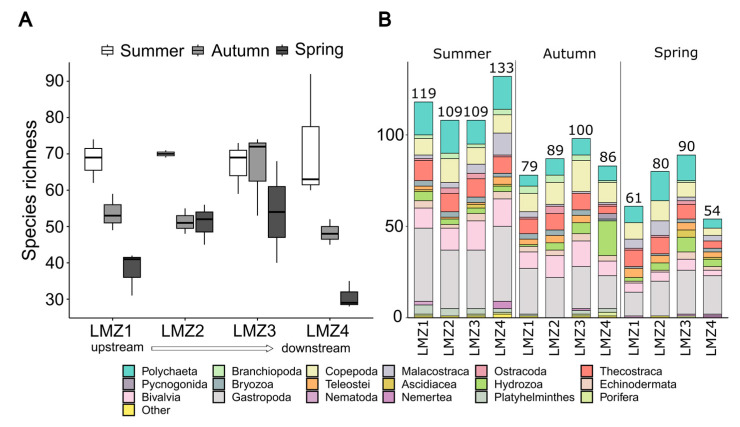
Seasonal and spatial influences on recovered zooplankton species richness (**A**) and taxonomic composition of the species recovered at all sites throughout the study duration (**B**). N = 3. In (**B**), data obtained in all replicates were joined together before analysis.

Overall, average species richness displayed a decreasing tendency through all the study periods (ANOVA, F = 27.9, *p* < 0.01), but, at LMZ2 and LMZ3, species richness displayed a different response to seasonal variation compared to that of LMZ1 and LMZ4, which declined through all seasons (Figure 3A). The trending decline in species richness attained was indeed observed at the intermediate sites: at LMZ2, a strong decline in species richness was observed from summer to autumn, but not from autumn to spring; while at LMZ3, the opposite pattern was observed: species richness slightly increased from summer to autumn but decreased in spring. Spatially, the recovered species richness showed an overall increment toward downstream peaking at LMZ3, followed by a steep decline at LMZ4, supported by Tukey’s post-hoc (*p* < 0.05, comparison between LMZ3 and LMZ4), which was a pattern observed more particularly in autumn and spring samples (Figure 3A).

Furthermore, the effect of seasonal and spatial variation diverged between different taxonomic groups, particularly those encompassing meroplankton. For instance, Polychaeta (Annelida) were more well represented during the summer and spring (in the latter at LMZ2 and LMZ3), while Mollusca were better represented in the summer, in particular Gastropoda (Figure 3B). Hydrozoa (Cnidaria) were recovered throughout the study duration; however, a greater number of species was observed in the LMZ4-autumn, which was characterized by the recovery of 19 species, of which 13 were exclusive to these samples (coincided with the 3 exclusive Pycnogonida records).

**Figure 4:**
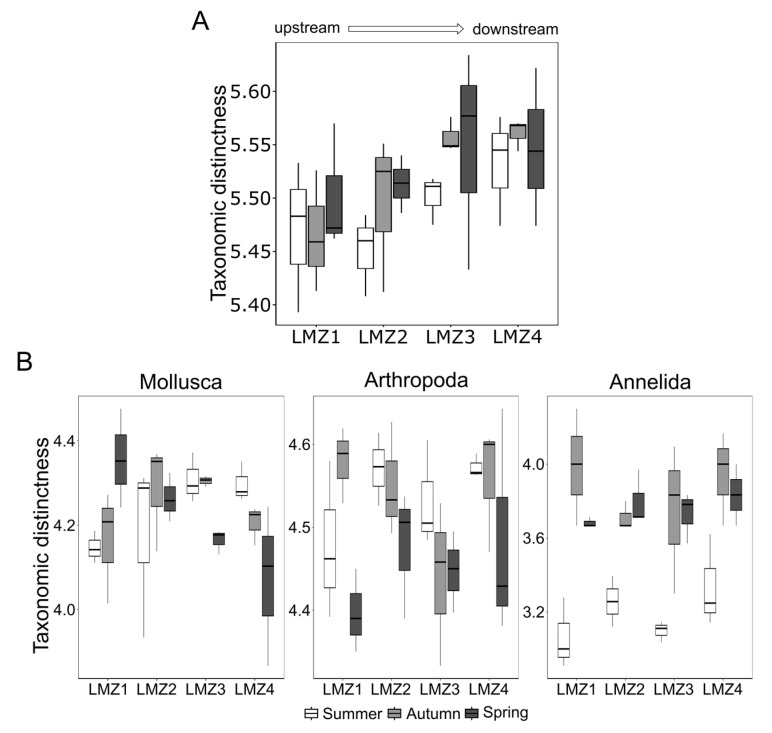
Seasonal and spatial influences on recovered zooplankton taxonomic distinctness (**A**), and group-specific taxonomic distinctness of the most relevant phyla based on species richness (Mollusca, Arthropoda and Annelida) (**B**).

On the other hand, the taxonomic distinctness demonstrated, in general, a different pattern from that observed for species richness. For instance, throughout the spatial gradient of the study area, a trending increase in taxonomic distinctness was observed from the upstream to downstream sites during all three sampled seasons (ANOVA, F = 3.16, *p* < 0.05) (Figure 4A), although no influence was found from season. Indeed, at every site, the taxonomic distinctness was maintained stably throughout the study duration. Nevertheless, the taxonomic distinctness was, in general, fairly stable, ranging from 5.39 (at LMZ1-summer) to 5.63 (at LMZ3-spring).

The taxa-specific taxonomic distinctness also demonstrated divergent patterns compared to the general analysis of zooplankton diversity (Figure 4B). Annelida and Arthropoda taxonomic distinctness was demonstrated to be more influenced by seasonal variation (ANOVA, F = 32.98, *p* < 0.01; F = 4.95, *p* < 0.05, respectively), but not by the estuary’s spatial gradient. However, both the Annelida and Arthropoda demonstrated diverging patterns from each other: the former displayed much lower values in the summer than in the remaining seasons, while the latter revealed a decreasing tendency throughout the study’s duration (from summer, autumn, and spring) (Figure 4B). Moreover, the Mollusca taxonomic distinctness revealed an opposite trend compared to that of the general zooplankton diversity, decreasing from LMZ1 toward LMZ4, but no significant differences were found in such variation nor from a seasonal effect (ANOVA, F = 0.18, *p* > 0.05; F = 0.64, *p* > 0.05, respectively). The taxonomic distinctness was indeed highly dependent on the taxonomic group analyzed, and varied differently for different taxonomic groups, e.g., for Annelida, the minimum and maximum taxonomic distinctness scored higher than 1, while for Arthropoda and Mollusca such an interval was much lower (Figure 4B).

**Figure 5:**
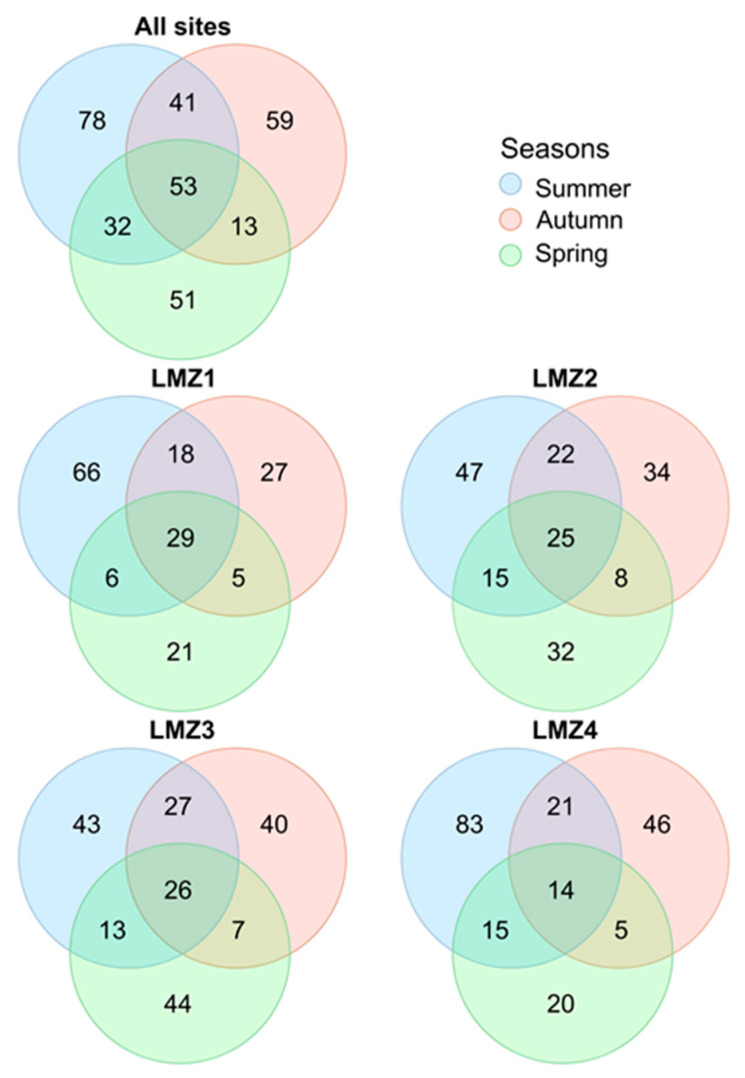
Seasonal partitioning of zooplankton diversity recovered with metabarcoding throughout the whole sampled spatial extension and for each sampling site: LMZ1, LMZ2, LMZ3, and LMZ4.

**Figure 6:**
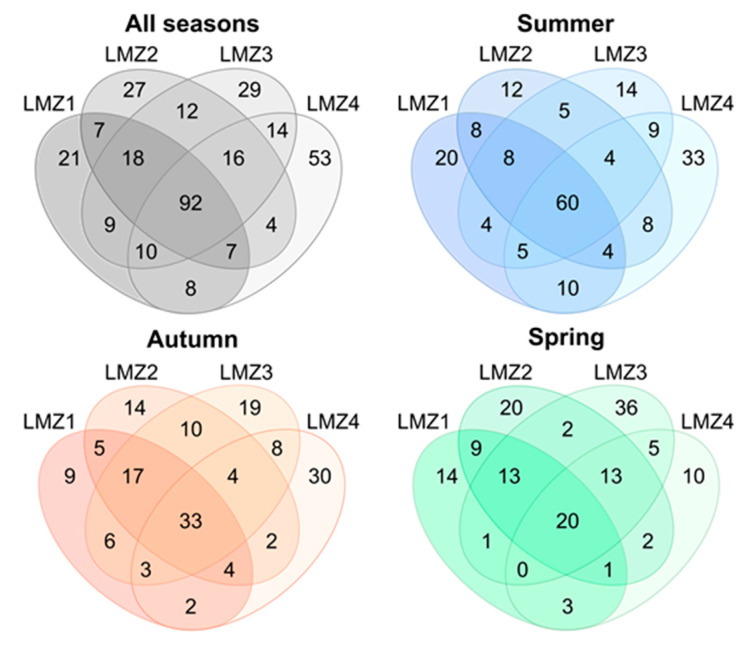
Spatial partitioning of zooplankton species recovered with metabarcoding throughout the study duration and for summer, autumn, and spring samples. From the most upstream site (LMZ1) to the most downstream (LMZ4).

The partitioning of the species across all seasons (in general and for each site, separately, Figure 5) and across all sites (in all seasons and for each season separately, Figure 6) is displayed in Venn diagrams. In general, only 53 species were recovered in all seasons and the highest number of exclusive species was detected in summer (78 species). The highest number of species was shared between summer and autumn (41 species), while the lowest number was shared between autumn and spring (13 species), and this pattern was observed throughout the estuary, at all sampled sites (Figure 5). In addition, the highest number of exclusive species was detected in summer (66, 47, and 83 species for LMZ1, LMZ2, and LMZ4) at all analyzed sites, with the exception of LMZ3, where the highest number of exclusive species was detected in spring (44 species). On the other hand, 92 species were detected at all sampled estuarine sites (60 in summer, 33 in autumn, and 20 in spring). The highest number of exclusive species was recovered at LMZ4 (53 species, approx. 16%), while the lowest was at LMZ1 (21 species) (Figure 6). The highest number of shared species was found between LMZ3 and LMZ4 in the summer and autumn (9 and 8 species, respectively); while in spring, the highest number of species (9 species) was shared between the most upstream sites (LMZ1 and LMZ2) (Figure 6).

### 3.4. Seasonal and Spatial Dynamics of Zooplankton Structure Composition

**Figure 7:**
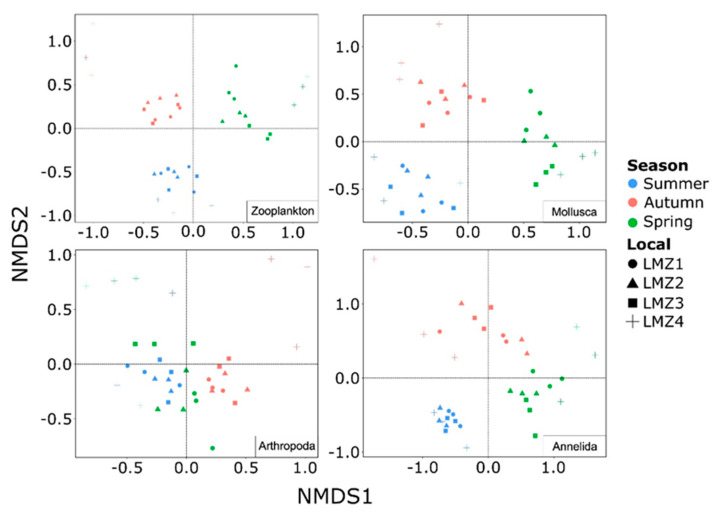
nMDS ordination of Lima estuary’s zooplankton recovered through metabarcoding employing a multi-marker approach (A; Stress = 0.13), and the most relevant phyla (Mollusca: Stress = 0.16; Arthropoda: Stress = 0.17; Annelida: Stress = 0.14). All similarity matrices used were based on presence–absence data (Jaccard’s dissimilarity index).

A non-metric multidimensional scaling analysis indicated that the zooplankton recovered from the Lima estuary were mostly structured by season, but considerable spatial turnover was also observed (Figure 7 and Table 1). Both the summer and spring samples displayed closer relations to the autumn cluster (NMDS2 and NMDS1, respectively) than with each other (pairwise PERMANOVA, *p* < 0.01), but still maintain their own characteristic compositions (pairwise PERMANOVA, *p* < 0.01 for both). LMZ4 was demonstrated to be the most species-level composition divergent site from the estuary, forming distinct clusters in all seasons (Figure 7), except in spring, where LMZ4 clustered with LMZ3 samples, thereby dividing the estuary into two different clusters (Figure 7). Still, in autumn, the most downstream location displayed the most divergent zooplankton composition from the study. Indeed, a high number of exclusive Hydrozoa and Pycnogonida were recovered, while Annelida contribution was lower in these samples (Figure 3B); however, the recovery of the Hydrozoa *Clytia gracilis* (Sars, 1851), *Clytia paulensis* (Vanhöffen, 1910), *Obelia longissima* (Pallas, 1766) and *Orthopyxis integra* (MacGillivray, 1842), the Pycnogonida *Achelia echinata* (Hodge, 1864), the Gastropoda *Trinchesia caerulea* (Montagu, 1804), the Annelida *Malacoceros fuliginosus* (Claparède, 1868), and the Chaetognatha *Parasagitta friderici* (Ritter-Záhony, 1911) were found to be significantly correlated to this ordination (*p* < 0.01) and better explained such a distribution (Table S5, Supplementary Material).

Furthermore, the species-level composition throughout the study differed between the taxonomic groups, with the exception of the Mollusca, for which the observed seasonal and spatial patterns were, indeed, very similar to those found for general zooplankton (Figure 7 and Table 1). Although summer and autumn samples displayed their own clustering (NMDS2), both were more related to each other than to the spring Mollusca zooplankton composition (NMDS1), though Mollusca composition along the estuary spatial gradient varied less. In both the autumn and spring clusters, the LMZ4 samples were closer to those from the remaining sites.

On the other hand, zooplankton’s Arthropoda and Annelida composition revealed differing patterns compared to the general zooplankton and Mollusca compositions (Figure 7). Arthropoda displayed distinct seasonal clustering, where summer and spring were highly similar to each other; however, a particular spring cluster was formed for NMDS1, encompassing the LMZ1-LMZ3 samples, and separating autumn from summer. An opposite pattern was observed for Annelida, where the autumn and spring clusters were more closely related to one another than to summer samples, although to a lesser extent. The LMZ4 species-level composition formed distinct clusters for both the Arthropoda and the Annelida, although within the latter a greater variation was displayed (Figure 7).

**Figure 8:**
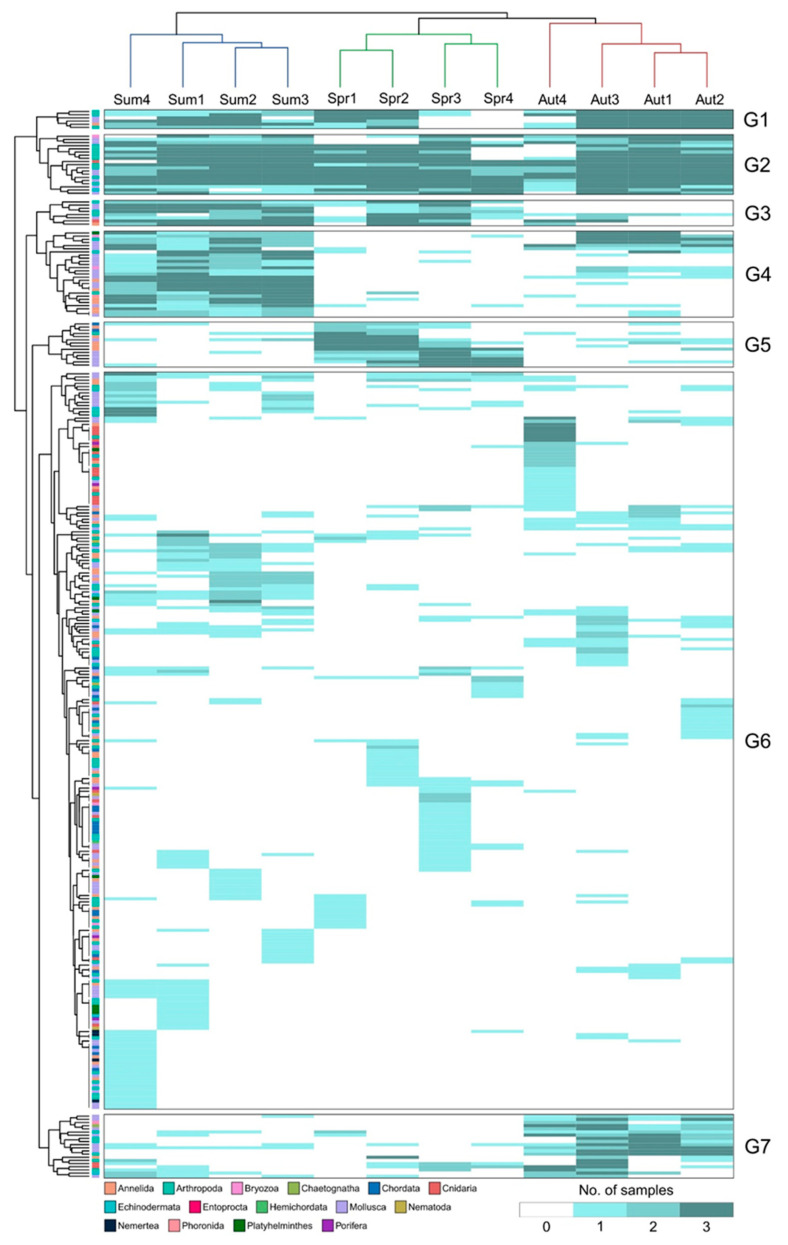
Species-level (y-axis) and sampling event (x-axis) clustering associated with a heatmap color-coded based on the number of replicates where each taxon was detected on each sampling event (0–3, with 0 indicating absence in all replicates and 3 presence in all replicates). Both dendrograms are color-coded based on each taxa phylum for the y-axis and based on the sampled seasons for the x-axis (blue = summer, green = spring, red = autumn).

Considering the relative abundance (number of samples recovered), species-level clustering generated consistent results with the nMDS for the whole dataset. The set of 327 zooplankton species revealed clustering in seven groups (G1-7), ranging from 6 to 233 species, that appeared to better explain the stronger seasonal variation over spatial influence on the Lima estuary’s zooplankton (Figure 8). Indeed, the G4 (27 species), G5 (14 species), and G7 (20 species) clustered species with greater representation in summer, autumn, and spring, respectively—although a G4-subgroup included summer/autumn related species—and G3 (8 species) better represented summer/autumn, while species found throughout the whole seasonal and spatial gradient, with a remarkable representation in all samples, were clustered within G1 (6 species) and G2 (19 species). G6 was the broadest cluster, which included 233 species, where sample-specific species and species with lower representation were clustered together.

Spatial variation also had an effect on species clustering. For instance, the two-part division of the estuary observed in the spring was particularly found to be explained by G5 sub-groups and G1, while G6 sub-groups may have also had a small influence. In the summer, LMZ4 was displayed as the outgroup (similarly seen in Figure 7), but not as far as observed in the autumn samples. Such was not shown to be associated with species shift, but in fact with variations in the relative abundance, more particularly in the G1 and G4 clusters. The high dissimilarity of LMZ4 in the autumn from the remaining sites above-mentioned further supported the influence of the exclusive recovery of several Hydrozoa and the Pycnogonida composing the G6 subgroup, as well as the loss and lower representation of several taxa throughout G1, G2, G4, and G7. For further details regarding clustering of species, see Table S6 and Figure S1 (Supplementary Material).

## 4. Discussion

Our study had three main outcomes: (i) We confirm the great utility of DNA metabarcoding for assessing the seasonal and spatial dynamics of zooplankton in a temperate estuary. (ii) The multi-marker approach allowed for a broader characterization of zooplankton diversity and allowed for the species-level identification of taxa that would otherwise be assigned to higher taxonomic ranks. (iii) We detected several NIS for the first time in the Lima estuary, demonstrating the utility of zooplankton metabarcoding for the early detection of NIS.

### 4.1. DNA Metabarcoding Performance in the Assessment of Zooplankton Species in the Lima Estuary

For conducting our analyses, we opted to use species-level assignments, which are crucial for mapping species occurrence and distribution and to compare to what has been found so far in the estuary. Although we are highly aware of the issues pertaining the completeness and reliability of current DNA barcode reference libraries [73], that can have a strong impact on the taxonomic characterization of using species-level assignments on DNA metabarcoding, we point out several reasons for our choice: (i) a strong effort has been conducted in filling DNA barcode reference libraries for the studied region [74,75,76,77], in developing informatic tools for auditing COI reference records that were used in the current study (BAGS; see [78]), and in curating reference libraries [76,79,80]; (ii) gap-analyses of compiled lists of historic zooplankton and macrozoobenthos species in the study indicated very acceptable COI and 18S coverage in both BOLD and GenBank (97%, for zooplankton species and 85.2% for macrozoobenthos) [81]; and (iii) the reliability of species-level taxonomic assignments has been a posteriori verified based on currently available data (66% of the detected species are considered reliable).

Zooplankton assessments in the Lima estuary have been scarce and generally lacking in thorough and species-level taxonomic characterization. In fact, to the best of our knowledge, only one study had previously characterized the zooplankton community’s structure in the Lima estuary, but with an emphasis on Copepoda diversity [16], while the remaining studies focused on either ichthyoplankton [42,82,83,84] or functional holoplankton and meroplankton abundance and biomass [85,86,87]. Thus, meroplankton characterization has been typically less resolved and overlooked through morphology-based analysis. Herein, metabarcoding-derived data revealed a greater diversity of planktonic metazoans occurring in the Lima estuary than what has been previously described from morphology-based historical reports [16,42,82,83,85,86,87]. A higher performance in biodiversity recovery was expected with DNA metabarcoding since its potential for uncovering unreported biodiversity, including that of meroplankton screening efficacy, has been shown in several other studies [31,33,88]. In addition, the recovered zooplankton from the Lima estuary have indeed improved the species-level identifications of meroplankton, such as Bivalvia, Gastropoda, Polychaeta, and Hydrozoa (Figure 2B), compared to previous morphology-based surveys, and further allowed the detection of additional occurring planktonic forms of taxa not yet reported in the estuary, such as Porifera [16,85] (Table S7, Supplementary Material).

However, the discordance between the herein-recovered taxa and historical records is indeed considerable. For instance, sampling effort (spatially and temporally) is one of the pivotal considerations taken into account in zooplankton characterization and it is likely that it may have influenced the resulting characterization—both spatial and temporal profiles of the current study did not fully overlap those from previous studies (i.e., the most recent study was six to seven years apart from sample collection in the current study). Species list compilation may also have influenced results, as only published studies were considered, whereas no input was considered from other sources, such as the Global Biodiversity Information Facility (GBIF) database, nor WoRMS. Furthermore, communities’ fluctuations may have also played a role in the observed gap with species’ introduction into/extinctions in the estuary. For instance, plankton periodically produce resting stages that sink and accumulate in the sediment. These diapausing stages are prevalent in estuaries and lagoons [89] and remain dormant for long periods of time before hatching. Thus, they may have played a role in the differences between results of our study and historical records. That is, the diversity of resting stages may be richer than the expressed one [90,91], including those of historical surveys and the current assessment through DNA metabarcoding. For instance, Rubino & Belmonte [91] reported 80 species in sediment samples, which were absent from plankton sampled from the water column.

Thus far, the uncovering of a high meroplankton diversity further supports the nursery role of the Lima estuary for several macrozoobenthos species. In fact, earlier zooplankton assessments demonstrated high meroplankton abundance/biomass in certain regions within the Lima estuary [85,86,87]. The hydrodynamics of the estuary may indeed play a larger role than what has been documented in previous assessments, despite the different sampling and identification methodologies employed [16,92]. In addition, the residence time and the semidiurnal and mesotidal regime of the Lima estuary generate highly suitable conditions for the development of a dominant stationary wave [84,93], which may allow enough time for the development of meroplankton inside the estuary [94]. Indeed, a higher abundance of several meroplankton groups in the Lima estuary has been reported than in the neighboring Minho estuary (average flushing rate of 300 m s^−1^), [16], which may have accounted for the considerably high meroplankton diversity found at the Lima estuary through metabarcoding. Furthermore, some studies have been pointing out that the Lima estuary supports several fish species, relevant to local fisheries, as a nursery zone [42,84,95]. However, herein ichthyoplankton recovery resulted in 20 fish species from which some assignments might be questionable, particularly in the 18S dataset, since the 18S rRNA gene is too conserved across a broad range of fish species [22].

### 4.2. COI and 18S rRNA Gene Markers Displayed Minute Overlap in Zooplankton Species Detection 

The taxa-overlap between both molecular markers was strong when considering the higher taxonomic ranks, but the more specific the identification the greater the complementarity found between both markers. Indeed, the proportion of exclusive detections with both markers was remarkably high, and only 23 species were taxonomically assigned in simultaneous COI and 18S reads. The high complementarity between the employed markers was expected, since previous studies have demonstrated a considerable range of exclusive detections for the species-level identification of marine zooplankton [30,41,52,88]. Generally, the mitochondrial COI has been used for more reliable species-level assignments of recovered reads and is the standard DNA barcode for the molecular-based identification of metazoans [96]—although at the cost of, e.g., the high prevalence of primers–templates mismatch leading to PCR associated bias [97,98]—while the nuclear 18S rRNA gene has been traditionally used in aquatic microbial eukaryote assessments [99,100], since it displays a broader taxonomic scope—hence, it has an extensive reference database. However, 18S sequences can be too conserved to discriminate organisms at low taxonomic levels, so it is usually used for broad-range high-rank taxonomic assignments [30,41,52,88]. In the present study, species-level assignments were not possible for 26,188 of the 18S reads; however, the COI allowed for greater resolution with species-level identifications of several 18S taxa that were only assigned to genus level, namely, *Balanus*, *Bougainvillia*, *Eubranchus*, *Diopatra*, *Harpacticus*, *Hydractinia*, *Polygordius*, and *Tomopteris*. Still, species-level identifications with 18S should be considered with caution [101,102].

### 4.3. DNA Metabarcoding Performance in the Detection of Non-Indigenous Species (NIS) in Zooplankton Samples

The complementarity of COI and 18S in species recovery was crucial for the detection of NIS. A high number of NIS (16 species, representing 4.9% of the total detected species) were recovered from multi-spatial and -seasonal sampling in the Lima estuary, where only *Pseudodiaptomus marinus* was detected with both markers. Most of the NIS previously reported, based on historical records, were not detected in the current study, namely *Acartia (Acanthacartia) tonsa* (Dansa, 1849), *Corbicula fluminea* (Müller, 1774), or *Mya arenaria* (Linnaeus, 1758). *Austrominius modestus* was the only NIS previously reported to occur in the Lima estuary (Table S7, Supplementary Material) and recovered through DNA metabarcoding in the current study. Therefore, DNA metabarcoding allowed for the detection of several NIS which appear to be new reports in the Lima estuary, all of which were documented in Portugal, in addition to those already reported from previous metabarcoding-based assessments in a more enclosed site of the estuary: the recreational marina of Viana do Castelo [103,104,105].

Additionally, several reads recovered from a singular sample were assigned as *Heniochus acuminatus* (Linnaeus, 1758), an Indo-Pacific fish and putative first introduction in the European Atlantic coast. However, morphological records are required for confirmation, but this species has been already documented as a non-indigenous species in both the southwestern Atlantic coast and in the Ukrainian coast of the Black Sea [106,107,108], and has been more recently reported in Spanish territory (Canary Islands) [109]. On the other hand, the Bryozoa *Watersipora* spp. was detected with COI, and this genus includes the *Watersipora subtorquata* (d’Orbigny, 1852) that has been reported as a NIS in Portugal, as well as the *Watersipora subatra* (Ortmann, 1890) which is a NIS in European waters, but species-level identifications were not possible with any of the genetic markers here employed. Hence, it is possible that either species was herein detected; however, further confirmation is required with, i.e., morphological assessments. The 18S reads further recovered *Balanus* sp. which COI resolved as *B. trigonus*. Furthermore, *Musculus lateralis* (Say, 1822) assigned reads were recovered with 18S, and a further revision posed it as a reliable identification based on current available data. This species is native to the Atlantic coast of North America and, to our knowledge, it has not yet been recognized as a NIS on European coasts, but a recent 18S-based metabarcoding assessment has also reported its presence on the coast of Sweeden; however, further morphological evidence is required to confirm its occurrence on European coasts [110].

Although *Mytilus* sp. COI and 18S reads here recovered were assigned as being *Mytilus edulis* (Linnaeus, 1758), both markers are unable to discriminate *Mytilus* spp. due to the unusual hybridization and biparental mtDNA inheritance [111,112]. Still, although it is not possible to confirm or deny the occurrence of *M. edulis*, such detections may in fact be considered the congener *Mytilus galloprovincialis* (Lamarck, 1819), since the distribution of both species has been well stipulated [113,114].

However, 18S NIS detections must be considered with caution. For further details on the reliability of the herein NIS detections, see Table S8 (Supplementary Material). Indeed, an assignment with an identity percent higher than 97% with COI leads, in general, to correct species identification (with a few exceptions, i.e., *Mytilus* sp.); whereas, in many cases, the assignment of 18S (even at 100% identity) may yield taxa not present in the studied areas due to the fact that related species included in the reference database may share exactly the same sequence for the 18S fragment used [115]. In the current study, three 18S-recovered NIS were considered unreliable assignments, namely the Bivalvia *M. mercenaria* and both Stolidobranchia (Ascidiacea) *M. squamiger* and *S. plicata* (Table S8, Supplementary Material); however, all of them have been reported on the Portuguese coast [116]. So, further effort is crucial in surveying the estuary, which, coupled with morphology-based assessment, would further improve the confirmation of the introduction and establishment of such species.

### 4.4. Species Richness, Taxonomic Distinctness, and Species Composition Influenced Primarly by Season and Secondarily by Within-Estuary Location

Complementarity between the markers was apparent throughout the study (Figure 2A) and consistent across the seasonal and spatial patterns of the recovered taxa, thus allowing us to attain a more complete picture of the Lima estuary’s zooplankton dynamics. Spatial patterns of zooplankton diversity were less evident than seasonal effects (Figure 6 and Figure 7), which are yet to be documented in the Lima estuary. However, similar conclusions were attained when considering abundance/biomass quantification [16,42,82,83].

The zooplankton spatial distribution is primarily associated with the geomorphology of the estuary (i.e., length, width, depth) that influences the hydrodynamics and concurrently physical/chemical and biological factors, which by itself promotes remarkable influence over zooplankton diversity disparity through the spatial gradient. However, seasonal changes are much more dynamic and relevant over zooplankton taxa by further having an effect over spatial parameters. Therefore, zooplankton richness distribution and composition were more strongly modulated by seasonal variation, while also presenting a considerable spatial shifting specific to sampled seasons throughout the estuary. A zooplankton survey downstream of Eiffel’s bridge, from 2010 to 2011, has shown abundance peaks during summer and a secondary peak during autumn [16], similar to zooplankton assessments from other sites at the Lima estuary [85,86,87], which are characteristic of temperate systems [16,117]. Such findings were comparable to those herein: higher zooplankton richness was observed in the summer and next autumn seasons (Figure 3A and Figure 5), as well as a greater zooplankton diversity and taxonomic representation at the river mouth (Figure 3A and Figure 6) [16]. However, the water parameters we measured also displayed considerable salinity and conductivity changes throughout the spatial gradient of the estuary (Table S1, Supplementary Material). This result was not that apparent in the community’s analysis; it is probably related to the fact that our water parameter measures were taken on the superficial portion of the water column, whereas the samples for the community analysis were from deeper layers.

Nevertheless, zooplankton richness peaking in summer is usually associated with previous winter floods with higher nutrients and sediment flows into the estuary [16] and accumulates in seasons of lower water currents, such as in the summer, which promote primary production and facilitate nursery areas [118], particularly as observed for meroplankton (e.g., gastropods and polychaetas) (Figure 3) [87]. Summer conditions have also been associated with greater ichthyoplankton representation [42], but our metabarcoding data showed higher richness in the spring. Autumn and spring patterns were demonstrated to be similar to previous abundance/biomass arrangements [85,86,87]. However, seasonal variation influenced several taxonomic groups differently, which shows that the taxa from different phyla responded differently to seasonal variation, but the majority highlighted the high dissimilarity of LMZ4 communities’ composition in comparison to remaining locations. Indeed, this study highlighted particular seasonal divergences between Arthropoda and Annelida and Mollusca and the general zooplankton patterns, indicating similar species-level compositions between summer/spring and autumn/spring for the former two taxonomic groups while the latter two shared higher similarity. For annelids, the taxonomic distinctness appeared to have supported species-level clustering (Figure 6), since the most related seasons demonstrated similar taxonomic distinctness as well. Still, the available data on the Lima estuary’s zooplankton is scarce, and partially and/or does not represent our sampling sites.

## 5. Conclusions

Overall, the present study provided a more resolved analysis of zooplankton occurring seasonally, spanning approximately 6 km of the Lima estuary, using a multi-marker DNA metabarcoding approach. Both the composition and species richness were demonstrated to be differently distributed due to seasonal variation and over the spatial gradient, although with a greater influence from the former. The autumn-occurring zooplankton from the most downstream site (LMZ4) of the estuary displayed the most unique composition, with several relevant species and high Hydrozoa and Pycnogonida richness; however, several dominant phyla responded differently to seasonal and spatial variation. Furthermore, the results demonstrated a high relevance of meroplankton in the zooplankton of the Lima estuary, something that has been also highlighted in previous studies, but with low taxonomic resolution. In addition, several NIS were detected which were not yet reported in previous morphology-based surveys of the Lima estuary, although already reported to occur in Portugal. These findings highlight the need for more studies on zooplankton composition in the estuary and surrounding areas. Such studies are crucial for the improvement of models and ecological quality assessments, supporting conservation and more sustainable ecosystem service management, and mitigating climate change’s effects on highly dynamic ecosystems, such as coastal ecosystems. 

## Figures and Tables

**Table 1:** Permutational analysis of variance (PERMANOVA) results of the most relevant taxonomic groups from the recovered zooplankton with DNA metabarcoding (999 permutations, based on Jaccard’s dissimilarity index).

Taxonomic Group	Season		Site	
	F	*p*	F	*p*
Zooplankton	9.57	<0.01	2.54	<0.01
Mollusca	10.91	<0.01	1.91	<0.01
Arthropoda	7.26	<0.01	3.97	<0.01
Annelida	11.18	<0.01	2.08	<0.01

## Data Availability

All data generated and analyzed during this study are included in this article and in its Supplementary Materials.

## Supplementary Materials

The following supporting information can be downloaded at: https://www.mdpi.com/article/10.3390/ani13243876/s1, Table S1: Measured physical and chemical parameters at each sampling event, and the specific coordinates for each sampling site; Table S2: Nanodrop results regarding DNA concentrations of all zooplankton samples; Table S3: Number of reads recovered from Illumina MiSeq sequencing and throughout the whole process of filtration, trimming, and taxonomic assignment until eligibility for analysis; Table S4: List of species and respective number of reads recovered with DNA metabarcoding from zooplankton samples using two molecular markers, COI and 18S. Non-indigenous species are marked with *;Table S5: Significant (*p* > 0.05) correlated species to both axis in the nMDS (NMDS1 and NMDS2) for the whole zooplankton recovered data; Table S6: Species list that clustered into 7 groups (G1-7); Table S7: Compiled list of zooplankton (Z) and macrozoobenthos (M) species reported from previous morphology-based studies, as well as markers which detected them in the current study; Table S8. List of the species detected in the current study, with associated marker recovery, and notes on the reliability of the detection; Figure S1: Species-level (y-axis) and sampling events (x-axis) clustering associated with a heatmap color-coded based on the number of replicates where each taxon was detected on each sampling event (0–3, with 0 indicating absence in all replicates and 3 presence in all replicates). Both dendrograms are color-coded based on each taxa phylum for the y-axis and based on the sampled seasons for the x-axis (blue = summer, green = spring, red = autumn). Cluster-specific species are found to the right of the heatmap; Info S1: Genoinseq amplification and sequencing report; Info S2: List of the reference libraries used for taxonomic assignment of COI reads (in order). Refs. [47,48,49,56,57,119,120,121] are cited in supplementary files.
