# Supplementary material for: Assessing the Seasonal and Spatial Dynamics of Zooplankton through DNA Metabarcoding in a Temperate Estuary
Source: Animals (Basel). 2023 Dec 16;13(24):3876. doi: 10.3390/ani13243876 (PMC10740712; doi:10.3390/ani13243876)
Supplement: Supplementary file 1 [file animals-13-03876-s001.zip › Moutinho et al_Supplementary_Material.pdf]

Supplementary Material to "Assessing seasonal and spatial dynamics of zooplankton through DNA metabarcoding in a temperate estuary on the NW of Portugal"

Info S1: Genoinseq amplification and sequencing report

Sample Preparation and Sequencing

Samples were prepared for Illumina Sequencing by 18S rRNA and COI gene amplification of the eukaryotic communities. The DNA was amplified for the hypervariable regions with specific primers and further reamplified in a limited-cycle PCR reaction to add sequencing adapters and dual indexes.

For the 18S V4 region, PCR reactions were performed for each sample using KAPA HiFi HotStart PCR Kit according to manufacturer suggestions, 0.3 μM of each PCR primer: forward primer TAReuk454FWD1 5'– CCAGCASCYGCGGTAATTCC -3' and reverse primer TAReukREV3 5'– ACTTTCGTTCTTGATYRA -3' (Lejzerowicz et al., 2015; Stoeck et al., 2010) and 2.5 μL of template DNA in a total volume of 25 μL. The PCR conditions involved a 3 min denaturation at 95 ºC, followed by 10 cycles of 98 ºC for 20 s, 57 ºC for 30 s and 72 ºC for 30 s, 25 cycles of 98 ºC for 20 s, 47 ºC for 30 s and 72 ºC for 30s and a final extension at 72 ºC for 5min.

For the COI region, PCR reactions were performed for each sample using KAPA HiFi HotStart PCR Kit according to manufacturer suggestions, 0.3 μM of each PCR primer: forward primer mlCOIintF 5'– GGWACWGGWTGAACWGTWTAYCCYCC-3' and reverse primer LoboR1 5'– TAAACYTCWGGRTGWCCRAARAAYCA -3' (Leray et al., 2013; Lobo et al., 2013) and 5 μL of template DNA in a total volume of 25 μL. The PCR conditions involved a 3 min denaturation at 95 ºC, followed by 35 cycles of 98 ºC for 20 s, 60 ºC for 30 s and 72 ºC for 30 s and a final extension at 72 ºC for 5 min. Negative PCR controls were included for all amplification procedures. The negative control samples sent by the client did not amplify for any primer pair.

Second PCR reactions added indexes and sequencing adapters to both ends of the amplified target region according to manufacturer's recommendations (Illumina, 2013). PCR products were then one-step purified and normalized using SequalPrep 96-well plate kit (ThermoFisher Scientific, Waltham, USA) (Comeau, Douglas, & Langille, 2017), pooled and pair-end sequenced in the Illumina MiSeq® sequencer with the MiSeq reagent Kit v3 (600 cycles), according to manufacturer's instructions (Illumina, San Diego, CA, USA) at Genoinseq (Cantanhede, Portugal).

Sequence data was processed at Genoinseq (Cantanhede, Portugal). Raw reads were extracted from Illumina MiSeq® System in fastq format and quality-filtered with PRINSEQ version 0.20.4 (Schmieder & Edwards, 2011) to remove sequencing adapters, trim bases with an average quality lower than Q25 in a window of 5 bases and eliminate reads with less than 100 bases for the 18S region and 150 bases for the COI gene. The forward and reverse reads were merged by overlapping paired-end reads with AdapterRemoval version 2.1.5 (Schubert, Lindgreen, & Orlando, 2016) using default parameters.

Results

The control samples ZEVP0, ZEOP0 and ZEPP0, did not produce amplicons and were not sequenced along with the samples.

References

Comeau, A. M., Douglas, G. M., & Langille, M. G. I. (2017). Microbiome Helper: a Custom and Streamlined Workflow for Microbiome Research. *MSystems*, *2*(1). https://doi.org/10.1128/mSystems.00127-16

Illumina. (2013). 16S Metagenomic Sequencing Library Preparation Preparing 16S Ribosomal RNA Gene Amplicons for the Illumina MiSeq System. *Illumina Technical Document*, (Part. No. 15044223 Rev. B.).

Lejzerowicz, F., Esling, P., Pillet, L., Wilding, T. A., Black, K. D., & Pawlowski, J. (2015). High-throughput sequencing and morphology perform equally well for benthic monitoring of marine ecosystems. *Scientific Reports*, *5*(August), 1–10. https://doi.org/10.1038/srep13932

Leray, M., Yang, J. Y., Meyer, C. P., Mills, S. C., Agudelo, N., Ranwez, V., … Machida, R. J. (2013). A new versatile primer set targeting a short fragment of the mitochondrial COI region for metabarcoding metazoan diversity: application for characterizing coral reef fish gut contents. *Frontiers in Zoology*, *10*(1), 34. https://doi.org/10.1186/1742-9994-10-34

Lobo, J., Costa, P. ., Teixeira, M. A., Ferreira, M. S., & Costa, M. H. C. and F. O. (2013). Enhanced primers for amplification of DNA barcodes from a broad range of marine metazoans Background: Building reference libraries of DNA barcodes is relatively, 1–8. https://doi.org/10.1186/1472-6785-13-34

Schmieder, R., & Edwards, R. (2011). Quality control and preprocessing of metagenomic datasets. *Bioinformatics*, *27*(6), 863–864. https://doi.org/10.1093/bioinformatics/btr026

Schubert, M., Lindgreen, S., & Orlando, L. (2016). AdapterRemoval v2: rapid adapter trimming, identification, and read merging Findings Background. *BMC Res Notes*, *9*. https://doi.org/10.1186/s13104-016-1900-2

Stoeck, T., Bass, D., Nebel, M., Christen, R., Jones, M. D. M., Breiner, H.-W., & Richards, T. A. (2010). Multiple marker parallel tag environmental DNA sequencing reveals a highly complex eukaryotic community in marine anoxic water. Molecular Ecology, 19, 21–31. https://doi.org/10.1111/j.1365-294X.2009.04480.x

Supplementary Material Info S2: List of the reference libraries used for taxonomic assignment of COI reads (in order)

1. SYS-MBRAVEC
2. SYS-CRLBACTERIA
3. SYS-CRLCHORDATA
4. SYS-CRLFUNGI
5. SYS-CRLINSECTA
6. SYS-CRLNONARTHINVERT
7. SYS-CRLNONINSECTARTH
8. SYS-CRLPROTISTA
9. DS-BIBLIO
10. DS-CRNS
11. DS-CRST
12. DS-CRUSNJ
13. DS-3150
14. DS-GAIMARIN
15. DS-EUROFISH
16. DS-POLYCHPT
17. DS-PTGB
18. DS-LTZPL
19. DS-MTANE
20. DS-MTANE2
21. DS-MTHD
22. DS-MTPD

The “SYS” prefix corresponds to the system’s reference libraries, while “DS” corresponds to all additional reference libraries. Underlined reference libraries correspond to personal barcoded and curated records in the Portuguese and Iberian coasts of several invertebrates’ groups. Additional reference libraries were selected in order of maximizing taxonomic assignment and detection of, i.e., putative non-indigenous species.
